# The sclerosing sertoli cell tumor of the testis: a case report

**DOI:** 10.1186/s13000-023-01351-7

**Published:** 2023-05-15

**Authors:** Xueyao Tang, Yifan Hu, Hong Zhou, Yang Zhou

**Affiliations:** 1 Department of Ultrasound, The Third People’s Hospital of Chengdu, Clinical College of Southwest Jiao Tong University, The Second Affiliated Chengdu Hospital of Chongqing Medical University, Chengdu, Sichuan China; 2 Department of Pathology, The Third People’s Hospital of Chengdu, Clinical College of Southwest Jiao Tong University, The Second Affiliated Chengdu Hospital of Chongqing Medical University, Chengdu, Sichuan China

**Keywords:** Sclerosing sertoli cell tumor, Sertoli cell tumor, Testicular neoplasm, Case report

## Abstract

**Background:**

Testicular Sertoli cell tumor (SCT) is very rare sex cord-gonadal stromal tumor, and sclerosing SCT (SSCT) is even rarer. So far, no more than 50 cases of SSCT have been reported. 80% of SSCTs are less than 2 cm in diameter, large volume mass is pretty unusual. SSCT is usually benign with very low malignant potential. However, it is easily misdiagnosed as a malignant tumor resulting in the removal of the entire testicle.

**Case presentation:**

A 55-year-old Chinese male patient presented with a six months’ history of right testis progressively enlargement and negative tumor markers. The physical examination was nothing special except for swelling in the right testicle. Imaging identified a large mass in right testicle with rich blood. A right radical orchiectomy was performed on suspicion of malignancy. However, the tumor was postoperatively diagnosed as SSCT, which pathologically consisted of a tubular pattern with regular nuclei and embedded in a densely collagenous stroma, as well as diffusely positive for vimentin, β-catenin and synaptophysin. After 7 months of follow up, no evidence of local recurrence and metastasis has been observed.

**Conclusion:**

This rare case is helpful to expand the knowledge of the testicular tumor and alert us fully understand the rare variant of SCTs in order to choose the optimal management when they encounter SSCT.

**Supplementary Information:**

The online version contains supplementary material available at 10.1186/s13000-023-01351-7.

## Introduction

SCT is very rare sex cord-gonadal stromal tumor, accounting for 0.4–1.5% of all primary testicular neoplasms [1]. Based on World Health Organization (WHO) Classification 2004, SCT is identified as three histological subtypes: Not Otherwise Specified (NOS), large-cell calcifying and sclerosing. Only 17% of SCTs are malignant [2]. Most SCTs fall into the category of SCT-NOS. SSCT, especially in larger size (80% less than 2 cm in diameter), is extremely rare. To date, less than 50 cases of SSCT have been reported since it was first described by Zukerberg in 1991 [3]. SSCT is generally regarded as benign, however, due to the absence of specificity in the symptoms, signs and auxiliary examinations, it is easy to be misdiagnosed preoperative. Histopathologic examination is the only method to make a definitive diagnosis. Herein, we present an additional case of SSCT in a 55-year-old Chinese male patient, review the relevant literature and discuss its clinical characteristics and treatment options, in order to carry out further research on this rare tumor.

### Case report

A 55-year-old Chinese male was referred to the urology department of our hospital with a six months’ history of right testis enlargement, which had worsened in the past few days. There were no specific symptoms of fever, dysuria, osphyalgia, hematuria and impotence. He denied history of cryptorchidism, testicular traumas, varicocele, or prior scrotal surgery. On physical examination, there were no lymphadenopathy, anaemia, or gynaecomastia except for the swelling in the right testis. Swelling was non-tender and hard. However, admission laboratory investigations were entirely normal, including tumor markers, such as eta-human chorionic gonadotropin (β-HCG) and alpha fetoprotein (AFP).

**Fig. 1:**
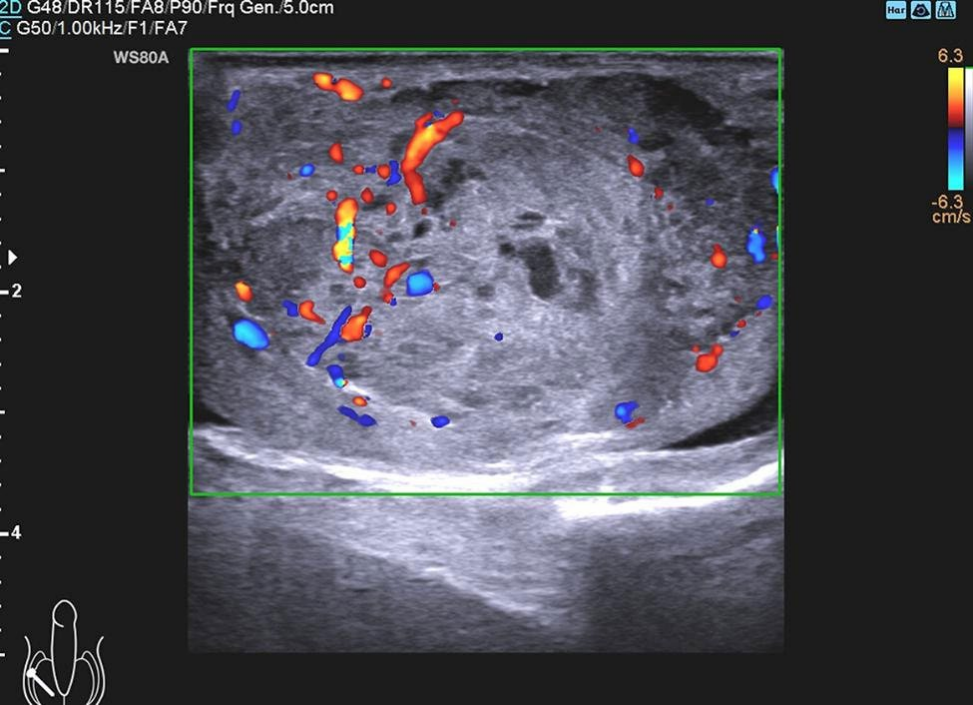
Ultrasonography showing a well-defined hypoechoic, heterogeneous mass in right testicle with internal rich flow on color Doppler.

**Fig. 2:**
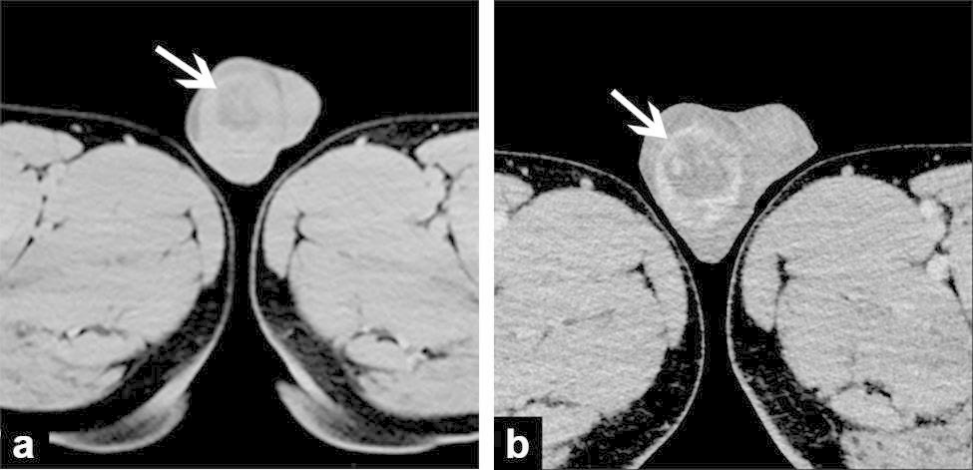
Computed tomography images. (a) plain and (b) enhanced CT showing a right testicular mass with heterogeneous density and enhancement

A scrotal ultrasound (US) examination revealed bilateral testicular hydrocele and a well-defined 4.9 × 3.0 × 4.1 cm hypoechoic, heterogeneous mass in the middle of right testicle with internal rich flow on color Doppler (Fig. 1). The left testicle, as well as the bilateral epididymis were normal. Given the large size, heterogeneous echo and abundant blood supply, the nodule was suspected to be malignant. Thus, the patient was sent for further work-up and evaluation with abdominal US and computed tomography (CT) scan. The results of chest CT and abdominal US were normal. On the pelvic CT images, there was a right testicular mass with heterogeneous density and enhancement, without pelvic lymphadenopathy or other sites of metastasis (Fig. 2).

**Fig. 3:**
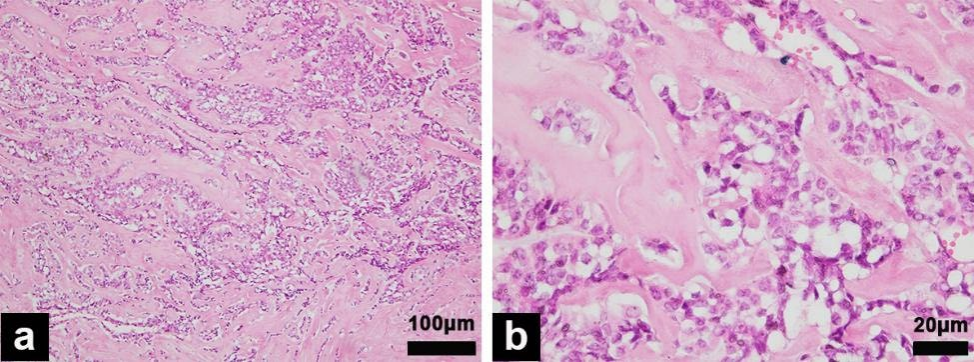
Histological images. (a) magnification ×100 and (b) magnification ×400 showing a tubular pattern with regular nuclei and embedded in a densely collagenous stroma

**Fig. 4:**
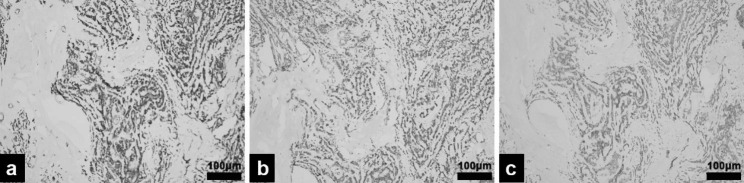
Immunohistochemical staining findings of SSCT. (a) vimentin postive. Magnification, ×100 (b) β-catenin positive. Magnification, ×100 (c) synaptophysin positive. Magnification, ×100

The patient underwent a right radical orchiectomy and the tumor measured 4.2 × 3.1 × 2.7 cm, without involvement of the adjacent spermatic cord or epididymis. The cut section was an intratesticular whitish-red firm nodule, mixed with hemorrhage and liquefactive necrosis. Microscopically, the neoplasm presented a tubular pattern with regular nuclei and embedded in a densely collagenous stroma (Fig. 3). No mitosis, or vascular invasion was detected to suggest malignant biological behavior. Immunohistochemical staining revealed the tumor cells are positive with vimentin, PCK, CD117, β-catenin, synaptophysin, S-100, P53 (Fig. 4), while negative with PLAP, Her-2, CD99, EMA, CR, α-inhibin, chromogranin A, Melan A. Ki-67 was 10%. The histomorphology and immunstains results favor the diagnosis of SSCT. The post-operative condition was stable. After 7 months of follow up, no evidence of local recurrence and metastasis has been observed by US and laboratory examination.

## Discussion

SSCT is an especially rare variant of SCTs which was first reported by Zuckerberg and colleagues in 1991 [3]. Said that, to date, less than 50 patients with SSCT have been reported in the literature. Reported cases with SSCT in testis exclusively occurs in postpubertal men between the age of 18 and 80 years, most commonly in middle age (mean, 45 y) [4]. All tumors presented unilaterally. About 11–25% of SCT-NOS have systemic symptoms due to estrogen secretion (feminization). Large-cell calcifying SCT was usually associated with endocrine disorders (masculinity and precocious puberty) or genetic syndromes. However, unlike the above two subtypes, no extragonadal clinical signs or symptoms due to excessive hormone production have been recorded in SSCT [5].

The patient was referred to our hospital with a high degree of suspicion of tumor because of his age and progressive enlargement of right testis. For scrotal swelling, the primary differential diagnosis would be inflammation, however, a normal blood routine and a lack of inflammatory signs, including skin thickening and redness, would be pretty atypical for this diagnosis. Other causes of scrotal swelling such as inguinal hernia or bilateral testicular hydrocele can be ruled out first by physical examination. Differentiation of benign and malignant mass at imaging is a crucial factor in identifying patients who require invasive tests and potential treatment for testicular lesions. However, at US SSCT often present as well-circumscribed hypoechoic lesions with prominent flow on color Doppler interrogation, which is non-specific and cannot make definitive diagnosis. Some studies also performed magnetic resonance imaging of SSCT and suggested that SSCT could be considered when T2-weighted images showed a distinct, well-defined, lobular hypointense mass with uniform enhancement in the testis [6, 7]. Detection of tumor serological markers is helpful in differential diagnosis. Until now, no patients with SSCT have elevated serum tumor markers or hormone dysfunction, but β-HCG is elevated in seminoma and choriocarcinoma, and ALP is elevated in most teratoma patients. Histopathologic examination is the only method to make a definitive diagnosis.

SSCT is typically small (80% less than 2 cm in diameter), but in our case, lesion size reach 4.2 cm, and even lesions greater than 7 cm have been reported previously [5]. Macroscopically, SSCT usually appear as a single, small, well demarcated, firm, yellow-whitish lesion in the testicular parenchyma. Histologically, in a typical tumor, the cells arranged in a pattern of solid nests, cords and tubules in a prominent sclerotic stroma. Since deposition of basement membrane or focal fibrosis is common in sertoli cell nodules and SCT, respectively, the WHO classification further narrowed the definition of SSCT to require that the collagen fiber should comprise at least 50% of the entire tumor [8]. SSCT diverged from SCT-NOS by its conspicuous, non-neoplastic, hypocellular fibrous stroma. The smaller cells and the lack of an intratubular component allows the exclusion of both the large cell calcifying and intratubular large cell hyalinizing variants. Moreover, calcification usually appear in the large-cell calcifying SCT in the form of amorphous masses, ossification and concentric masses. The absence of Reinke crystals and the presence of tubule formation support a diagnosis of SSCT rather than Leydig cell tumor. The highly heterotypic cells and focally distributed tubular structures aid differentiate spermatogonia from SSCT.

To distinguish SSCT from other potential mimics, the immunohistochemical stains are utilized for an accurate diagnosis. SSCT is characterized by positive vimentin, synaptophysin, CD56 [9]. 60% of SSCTs are positive for chromogranin A [4] .The expression frequency of inhibin in SSCT is low [3, 10]. The absence of inhibin staining (as in our case) that is the characteristics of Leydig cell tumor can serve to rule out this diagnosis as well. Negative reactivity for CD30, CD117, AFP, OCT4, and PLAP argues forcefully against the diagnosis of most germ cell tumors. A recent study of numerous cases verified the diagnostic value of nuclear β-catenin as it is frequently expressed in SSCT, whereas negative in large cell calcified SCT, granulosa cell tumor, unclassified SSCT, or stromal cell tumor [11]. These findings are compatible with our results. In our case, the Ki-67 index was 10%, which is generally above 30% in malignant tumors.

Most SSCTs are benign and have a significantly better prognosis than SCT-NOS and large cell calcifying. So far, only 2 malignant cases have been reported. One patient proved clinically malignant, showing bone metastases and died after 27 months after presentation [4].The other was histologically malignant, displaying cellular atypia, rete testis, frequent mitosis, and epididymis infiltration [3]. In view of malignant behavior in SSCT is rare, no criteria have been made to assist in the recognition of malignant mass. Such criteria, nevertheless, are established for SCT-NOS and include: large size (≥ 5 cm), increased mitotic activity, necrosis, nuclear atypia (moderate to severe), lymphovascular invasion, and infiltrative growth pattern [12, 13]. These established clinicopathologic criteria can be applied and help unfavourable behavior of individual SSCT with more or less accurately. No malignant feature was present in our case. Given the rarity of the tumor and large size of the tumor, the patient was scheduled for regular follow up after surgery.

In terms of treatment, the majority of the cases reported previously have undergone orchiectomy. However, whether fertility and sexual function are affected after surgical treatment also deserve surgeon’s attention, especially for such a tumor with high survival rate and low recurrence rate. Thus, the testicle-sparing surgery should be strongly recommended for patients with small bilateral lesions. When the intraoperative frozen section analysis is uncertain, the surgeon should reserve the testis and awaits a final pathology consultation. Secondary orchiectomy can be proceeded if the subsequent pathology indicates a non-stromal tumor.

SSCT was regarded a separate entity until the 2016 revision of the WHO classification, where it was specified a morphologic variant of SCT-NOS due to the similar clinical features and same molecular features including CCNB1 gene mutation, nuclear staining of β-catenin protein (> 70%) and diffuse positive expression of cyclin D1, in contrast to SCT-NOS [14, 15]. In the current classification, intratubular large-cell hyalinizing sertoli cell neoplasia has been identified as a separate entity under SCTs [8]. Noticeable, in addition to the extent of associated non-neoplastic stroma, there were differences in inhibin, S100 protein, androgen receptor and PAX2/PAX8 cocktail expression between SCT-NOS and SSCT [16]. Therefore, it is recommended to designated the previous SSCT as a sclerosing pattern of SCT-NOS.

In conclusion, SSCT is very rare and usually manifests as a palpable mass without malignant behavior in a postpubertal man. Although no specific imaging characteristics for SSCT were reported, and serum tumor markers or hormone levels are normal, we believe the testicle-sparing surgery or a second look surgery are achievable with the collaboration of pathologists and surgeons, which is particularly beneficial for patients with fertility needs.

## Electronic supplementary material

Below is the link to the electronic supplementary material.

Supplementary Material 1

Supplementary Material 2

## Data Availability

The original contributions presented in the study are included in the article/Supplementary Material, further inquiries can be directed to the corresponding author.

## Abbreviations

SCT: Sertoli cell tumor
SSCT: Sclerosing Sertoli cell tumor
WHO: World Health Organization
NOS: Not Otherwise Specified
β-HCG: Eta-human chorionic gonadotropin
AFP: Alpha fetoprotein
US: Ultrasound
CT: computed tomography
