# Supplementary figures and images for: The sclerosing sertoli cell tumor of the testis: a case report
Source: Diagn Pathol. 2023 May 15;18:61. doi: 10.1186/s13000-023-01351-7 (PMC10186805; doi:10.1186/s13000-023-01351-7)

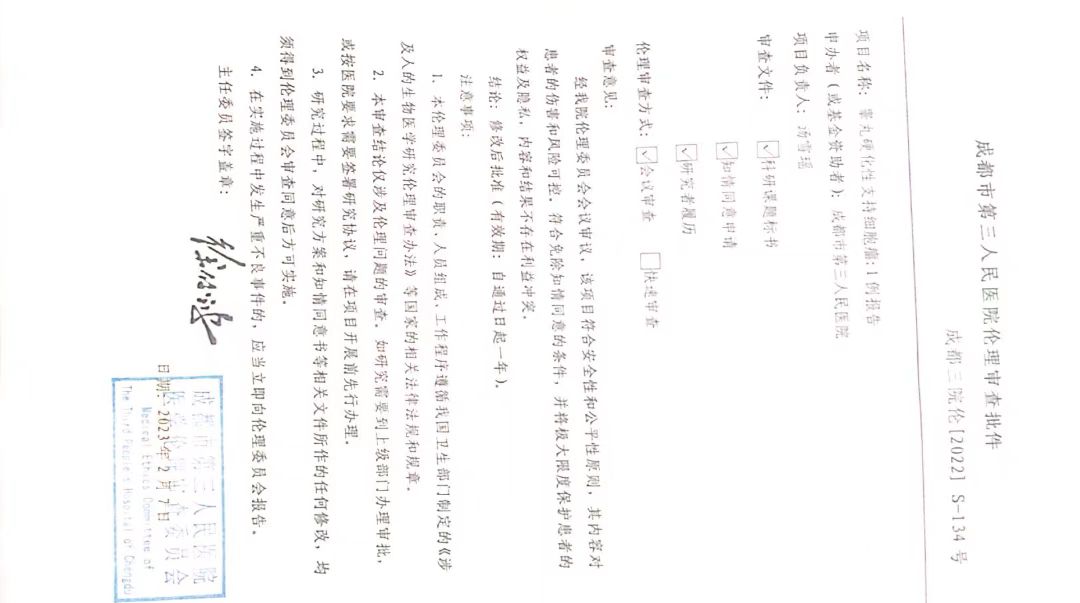

Supplement: Supplementary file 1 — Supplementary Material 1 [file 13000_2023_1351_MOESM1_ESM.jpg]
