# Supplementary material for: The sclerosing sertoli cell tumor of the testis: a case report
Source: Diagn Pathol. 2023 May 15;18:61. doi: 10.1186/s13000-023-01351-7 (PMC10186805; doi:10.1186/s13000-023-01351-7)
Supplement: Supplementary file 2 — Supplementary Material 2 [file 13000_2023_1351_MOESM2_ESM.doc]

**知情同意书**

尊敬的患者，您好：

经入院后相关检查，您行了右侧睾丸切除术，根据病理结果，考虑您的右侧睾丸肿物为硬化性支持细胞瘤。这种病例国际上报道很少，不到100例，很罕见，我们希望发表相关内容，增强医师对该疾病的理解。发表的相关文章内会隐去您的私人信息，但会交代整个发病过程级治疗转归，还会使用您的超声，CT，HE染色和免疫组化图片。若您同意，可签署此知情同意书。

医师：

日期：
